# Different packing motifs in the crystal structures of three mol­ecular salts containing the 2-amino-5-carb­oxy­anilinium cation: C_7_H_9_N_2_O_2_^+^·Cl^−^, C_7_H_9_N_2_O_2_^+^·Br^−^ and C_7_H_9_N_2_O_2_^+^·NO_3_^−^·H_2_O

**DOI:** 10.1107/S2056989020003163

**Published:** 2020-03-13

**Authors:** Edson T. Mukombiwa, William T A Harrison

**Affiliations:** a Department of Chemistry, University of Aberdeen, Meston Walk, Aberdeen AB24 3UE, Scotland

**Keywords:** benzoic acid, mol­ecular salt, hydrogen bonds, Hirshfeld surface, crystal structure

## Abstract

The syntheses and crystal structures of three mol­ecular salts of protonated 3,4-di­amino­benzoic acid, *viz*. 2-amino-5-carb­oxy­anilinium chloride, C_7_H_9_N_2_O_2_^+^·Cl^−^, (I), 2-amino-5-carb­oxy­anilinium bromide, C_7_H_9_N_2_O_2_^+^·Br^−^, (II), and 2-amino-5-carb­oxy­anilinium nitrate monohydrate, C_7_H_9_N_2_O_2_^+^·NO_3_^−^·H_2_O, (III), are described. The cation is protonated at the *meta*-N atom (with respect to the carb­oxy group) in each case. In the crystal of (I), carb­oxy­lic acid inversion dimers linked by pairwise O—H⋯O hydrogen bonds are seen and each N—H group forms a hydrogen bond to a chloride ion to result in (100) undulating layers of chloride ions bridged by the inversion dimers into a three-dimensional network. The extended structure of (II) features O—H⋯Br, N—H⋯Br and N—H⋯O hydrogen bonds: the last of these generates *C*(7) chains of cations. Overall, the packing in (II) features undulating (100) sheets of bromide ions alternating with the organic cations. Inter­molecular inter­actions in the crystal of (III) include O—H⋯O, O—H⋯(O,O), N—H⋯O, N—H⋯N and O—H⋯N links. The cations are linked into (001) sheets, and the nitrate ions and water mol­ecules form undulating chains. Taken together, alternating (001) slabs of organic cations plus anions/water mol­ecules result. Hirshfeld surfaces and fingerprint plots were generated to give further insight into the inter­molecular inter­actions in these structures. The crystal used for the data collection of (II) was twinned by rotation about [100] in reciprocal space in a 0.4896 (15):0.5104 (15) ratio.

## Abstract 2

The title mol­ecular salts containing the same cation accompanied by different simple anions show quite different inter­molecular connectivities.

## Chemical context   

The benzoate anion, C_7_H_5_O_2_^−^ is a classic ligand in coordin­ation chemistry, with over 1500 crystal structures reported in the Cambridge Structural Database (version 5.40, updated to February 2020; Groom *et al.*, 2016 ▸) for benzoate complexes of first-row transition metals alone. Functionalized benzoic acid derivatives add further structural variety: for example, –NH_2_ substituents at the *ortho*, *meta* and/or *para* positions of the benzene ring can form or accept hydrogen bonds with respect to nearby acceptor or donor groups and/or bond as Lewis bases to another metal ion (*i.e*. as a μ^2^-*N*,*O* or μ^3^-*N*,*O*,*O* bridging ligand). It should be noted that the presence of amine groups allows for protonation and the possible formation of mol­ecular salts with the amino­benzoic acid acting as the cation.

As part of our ongoing studies in this area (Khosa *et al.*, 2015 ▸), we now describe the syntheses and structures of three mol­ecular salts of protonated 3,4-di­amino­benzoic acid, *viz*. C_7_H_9_N_2_O_2_·Cl (I), C_7_H_9_N_2_O_2_·Br (II) and C_7_H_9_N_2_O_2_·NO_3_·H_2_O (III). Hirshfeld surface analyses have been performed to gain further insight into the inter­molecular inter­actions.
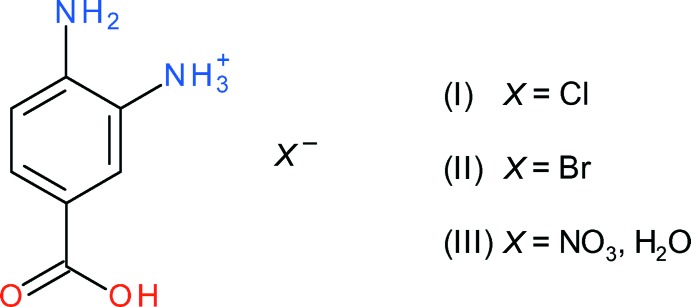


## Structural commentary   

**Figure 1:**
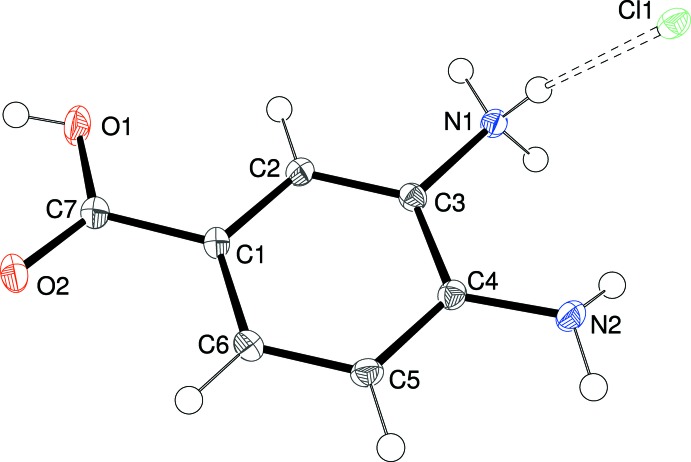
The mol­ecular structure of (I) showing 50% displacement ellipsoids. The N—H⋯Cl hydrogen bond is indicated by a double-dashed line.

**Figure 2:**
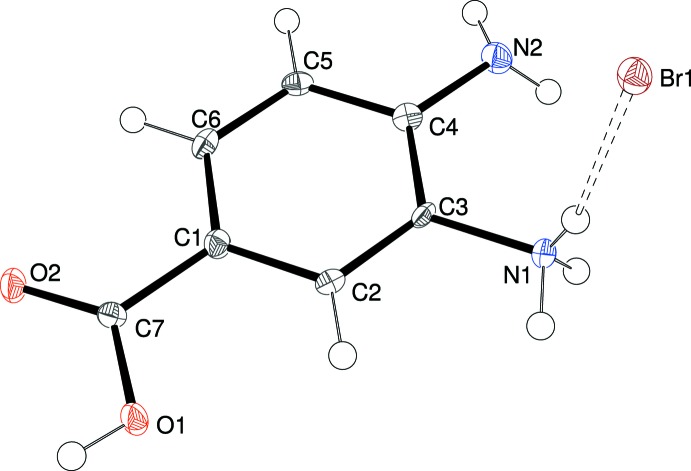
The mol­ecular structure of (II) showing 50% displacement ellipsoids. The N—H⋯Br hydrogen bond is indicated by a double-dashed line.

**Figure 3:**
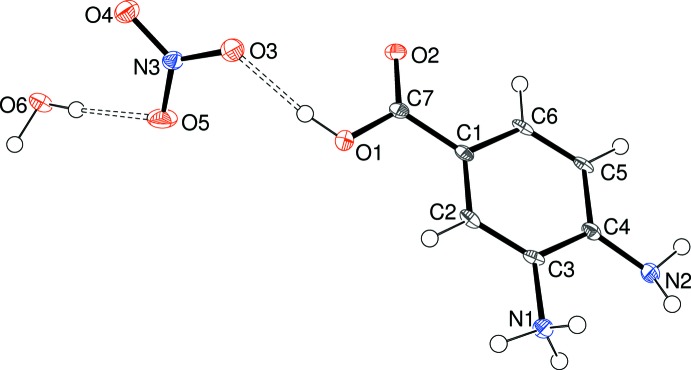
The mol­ecular structure of (III) showing 50% displacement ellipsoids. The O—H⋯O hydrogen bonds are indicated by double-dashed lines.

The contents of the asymmetric units of (I) (Fig. 1 ▸), (II) (Fig. 2 ▸) and (III) (Fig. 3 ▸) confirm them to be mol­ecular salts of 3,4-di­amino­benzoic acid (*i.e*. the C_7_H_9_N_2_O_2_^+^ 2-amino-5-carb­oxy­anilinium cation has been formed) and the appropriate strong acid (hydro­chloric acid, hydro­bromic acid and nitric acid, respectively); compound (III) also includes a water mol­ecule of crystallization. The neutral organic mol­ecule (C_7_H_8_N_2_O_2_) is known to crystallize as a zwitterion (Rzaczyńska *et al.*, 2000 ▸) with nominal intra­molecular proton transfer from the carb­oxy­lic acid to the *meta*-N atom and presumably exists in the same form in solution, thus the formal acid–base reaction to form the title salts involves proton transfer from the strong acid to the –CO_2_^−^ carboxyl­ate group of zwitterionic C_7_H_8_N_2_O_2_ to form a –CO_2_H carb­oxy­lic acid group; atom N1 remains protonated, to result in the C_7_H_9_N_2_O_2_^+^ cation.

The preference for the *meta* –NH_2_ group to be protonated in these salts compared to the *para* –NH_2_ group can be rationalized in terms of the potential loss of conjugation of the *para*-N-atom lone pair of electrons with the carb­oxy­lic acid grouping *via* the benzene ring, *i.e*., a small contribution of a quinoid (C=N^+^ containing) resonance form to the structure (Lai & Marsh, 1967 ▸): the mean bond lengths for C1—C2, C3—C4, C4—C5 and C1—C6 (single bonds in the quinoid structure) and C2—C3 and C5—C6 (double bonds) are 1.401/1.380, 1.400/1.381 and 1.403/1.373 Å for (I), (II) and (III), respectively (global averages = 1.401/1.378 Å). These data compare very well to the equivalent values of 1.399/1.375 Å established over 50 years ago from Weissenberg data for *p*-amino­benzoic acid (Lai & Marsh, 1967 ▸).

This electronic effect is also no doubt reflected in the fact that the C4—N2 (*para*) bond in the title compounds is notably shorter than the C3—N1 (*meta*) bond [distances in (I) = 1.378 (2) and 1.4640 (19), respectively; (II) = 1.387 (6) and 1.468 (6); (III) = 1.386 (5) and 1.457 (5) Å]. Even so, it may be noted that the bond-angle sums about N2 are 348.0, 340.4, and 339.2° for (I), (II) and (III), respectively, suggesting a tendency towards *sp* ^3^ hybridization (and presumably lone-pair localization) for the nitro­gen atom in each case: it also correlates with the fact that N2 accepts a hydrogen bond in the crystal of (III) (*vide infra*).

For each structure, the carb­oxy­lic acid group shows the expected clear distinction between the C7—O1H [(I) = 1.296 (2), (II) = 1.326 (5), (III) = 1.323 (6)Å] and C7=O2 [(I) = 1.252 (2), (II) = 1.216 (5), (III) = 1.232 (5) Å] bond lengths. The degree of twist of the –CO_2_H group with respect to the benzene ring is similar in the three salts [the angles between the mean planes passing through atoms C1–C6 and O1/O2/C7 are (I) = 6.39 (16), (II) = 0.5 (4), (III) = 3.8 (5)°].

## Supra­molecular features   

**Figure 4:**
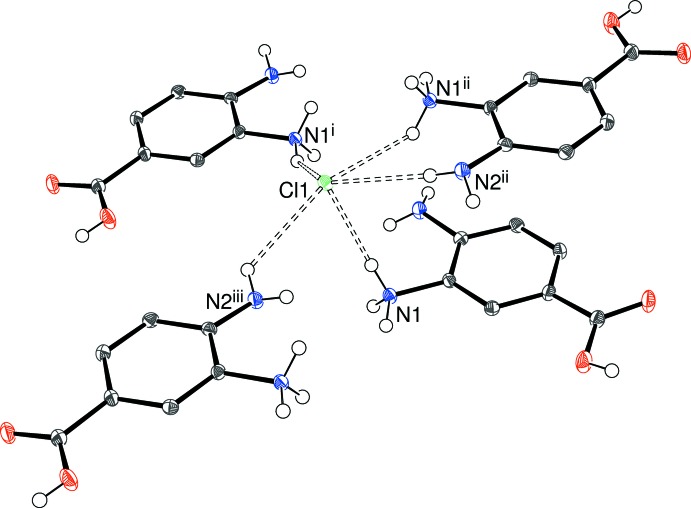
Environment of the chloride ion in the structure of (I) with N—H⋯Cl hydrogen bonds indicated by double-dashed lines. Symmetry codes: (i) 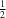 − *x*, *y* − 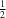, 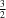 − *z*; (ii) *x*, 1 − *y*, *z* − 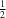; (iii) 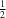 − *x*, 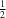 + *y*, 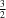 − *z*.

**Figure 5:**
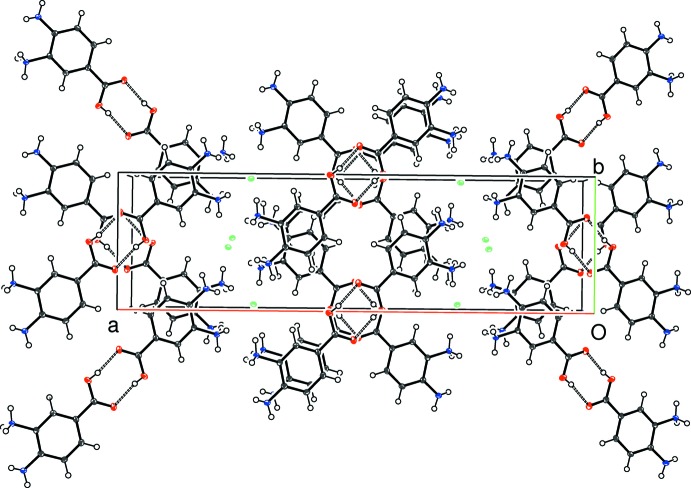
The packing for (I) viewed down [001]. The hydrogen bonds linking the carb­oxy­lic-acid inversion dimers are shown as double-dashed lines.

In the crystal of (I), the cations are connected into carb­oxy­lic acid inversion dimers *via* pairwise O—H⋯O hydrogen bonds (Table 1 ▸), thereby generating classical 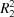
(8) loops. All five N—H groups link to a nearby chloride ion: the H⋯Cl contacts from the protonated –N1H_3_^+^ moiety (mean = 2.35 Å) are substanti­ally shorter than those arising from the unprotonated –N2H_2_ group (mean = 2.70 Å). As a result, the chloride ion accepts five N—H⋯Cl bonds from four cations (one cation bonds from both N1 and N2) in an irregular geometry (Fig. 4 ▸). The overall packing for (I) results in corrugated (100) sheets of chloride ions bridged by the carb­oxy­lic acid dimers into a three-dimensional supra­molecular network (Fig. 5 ▸).

**Figure 6:**
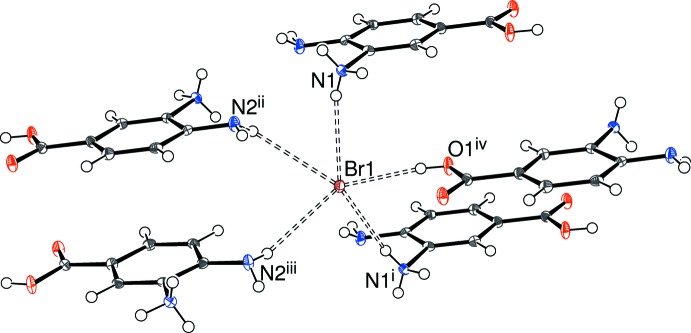
Environment of the bromide ion in the structure of (II) with N—H⋯Br and O—H⋯Br hydrogen bonds indicated by double-dashed lines. Symmetry codes: (i) *x*, *y*, *z* − 1; (ii) 2 − *x*, 1 − *y*, 1 − *z*; (iii) 2 − *x*, 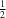 + *y*, 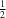 − *z*; (iv) 1 − *x*, 1 − *y*, −*z*.

**Figure 7:**
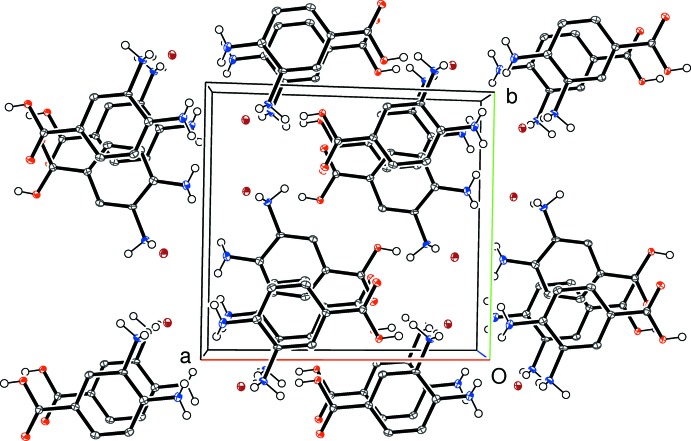
The packing for (II) viewed down [001].

Rather than carb­oxy­lic acid inversion dimers, the packing for (II) features O—H⋯Br hydrogen bonds as well as N—H⋯Br and N—H⋯O contacts (Table 2 ▸). The bromide ion (Fig. 6 ▸) is five-coordinated in an irregular geometry by four N—H⋯Br and one O—H⋯Br link arising from five different cations. The N1—H2*N*⋯O2 inter­action from the protonated –NH_3_^+^ group to the C=O bond of the carb­oxy­lic acid generates [010] *C*(7) chains of cations, with adjacent ions in the chain related by the 2_1_ screw axis. When all the hydrogen bonds are considered together, the packing for (II) can be described as a three-dimensional supra­molecular network of undulating (100) sheets of bromide ions alternating with the organic cations (Fig. 7 ▸). A notably short C—H⋯O inter­action (H⋯O = 2.23 Å), which reinforces the *C*(7) chain of cations, is also observed.

**Figure 8:**
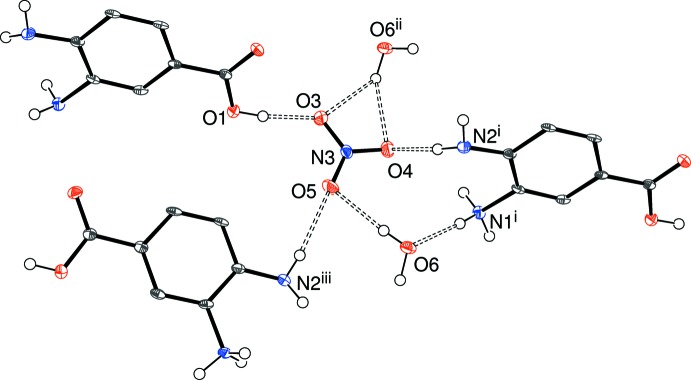
Environment of the nitrate ion in the structure of (III) with N—H⋯O and O—H⋯O hydrogen bonds indicated by double-dashed lines. Symmetry codes: (i) 1 + *x*, *y*, 1 + *z*; (ii) 2 − *x*, 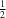 + *y*, 2 − *z*; (iii) −*x*, *y* − 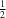; 1 − *z*.

**Figure 9:**
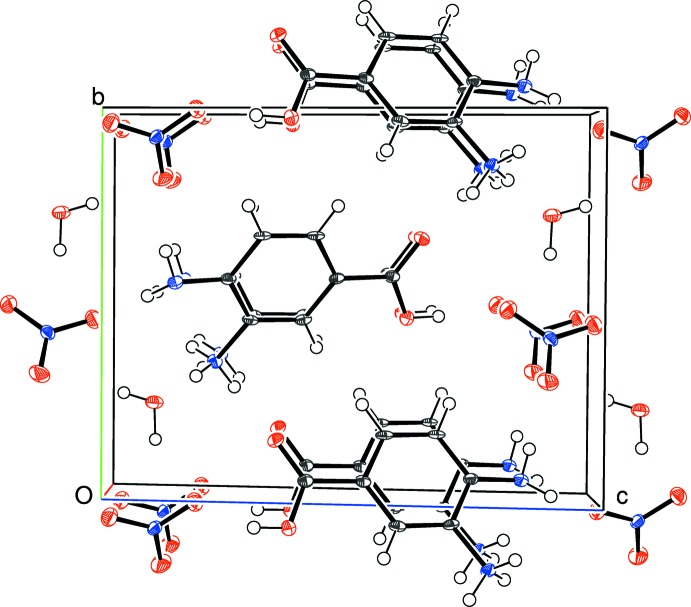
The packing for (III) viewed down [100].

The directional inter­molecular inter­actions in (III) (Table 3 ▸) include O_c_—H⋯O_n_, O_w_—H⋯(O_n_,O_n_), N—H⋯O_c_, N—H⋯N and N—H⋯O_n_ (c = carb­oxy­lic acid, n = nitrate, w = water) hydrogen bonds. The O atoms of the nitrate ion collectively accept four simple and one bifurcated hydrogen bond (Fig. 8 ▸) from three cations and two water mol­ecules. As in (II), an N1—H2*N*⋯O2 hydrogen bond in (III) generates *C*(7) chains of cations propagating in [010] with adjacent ions related by the screw axis but an N1—H3*N*⋯N2 inter­action also occurs; by itself it leads to [100] *C*(5) chains with adjacent ions related by translation; together, (001) hydrogen-bonded sheets of cations arise. When the nitrate ion and water mol­ecules are taken together, undulating hydrogen-bonded chains propagating in the [010] direction arise. Collectively, the packing in (III) (Fig. 9 ▸) can be described as alternating (001) slabs of nitrate anions + water mol­ecules and organic cations arising from a three-dimensional supra­molecular network of hydrogen bonds.

The three structures feature weak aromatic π–π stacking (Table 4 ▸). In each case, infinite stacks of mol­ecules, with considerable slippage between adjacent benzene rings, arise: these stacks propagate in the [001], [001] and [100] directions for (I), (II) and (III), respectively. A crystallographic *c*-glide generates the stacks in (I) and (II), whereas in (III) adjacent mol­ecules are related by simple translation.

## Hirshfeld surface analyses   

In order to gain further insight into the inter­molecular inter­actions in (I), (II) and (III), their Hirshfeld surfaces and two-dimensional fingerprint plots were calculated using *CrystalExplorer* (Turner *et al.*, 2017 ▸) following the approach recently described by Tan *et al.* (2019 ▸). The Hirshfeld surfaces of the cations in (I), (II) and (III) (see supplementary materials) show the expected red spots of varying intensity corresponding to close contacts resulting from the hydrogen bonds described above. The percentage contributions of the different type of contacts to the surfaces (Table 5 ▸) call for some comment. Despite their different packing motifs, especially the presence of pairwise O—H⋯O hydrogen bonds in (I) and O—H⋯Br and N—H⋯O inter­actions in (II), the contact percentages for the C_7_H_9_N_2_O_2_^+^ cations in (I) and (II) are strikingly similar, being mostly within 1% of each other. It may be seen that H⋯H (van der Waals) contacts dominate, followed by H⋯*X* (*X* = Cl, Br), H⋯O (donor) and then O⋯H (acceptor), with other contacts playing a minor role. The contact percentage data for (III) are decidedly different with H⋯O (donor) (32.2%) dominating and H⋯H (23.3%) relegated to second place, followed by O⋯H (acceptor) (12.6%). Despite the N—H⋯N hydrogen bond in (III), the H⋯N contact percentages barely differ for the three compounds. The contact percentages for the anions in (I) and (II) (Table 5 ▸) show them to be essentially ‘saturated’ by their hydrogen bonds, despite the irregular coordination geometries.

**Figure 10:**
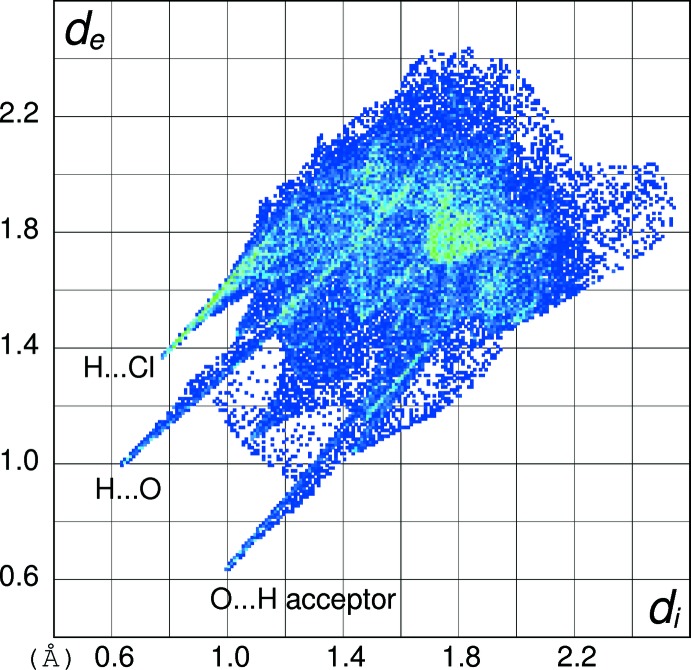
Hirshfeld fingerprint plot for the C_7_H_9_N_2_O_2_^+^ cation in (I).

**Figure 11:**
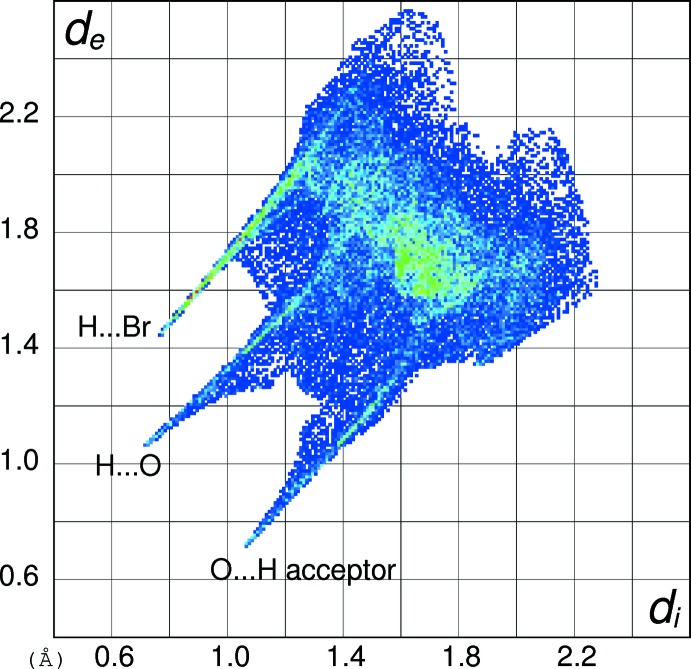
Hirshfeld fingerprint plot for the C_7_H_9_N_2_O_2_^+^ cation in (II).

**Figure 12:**
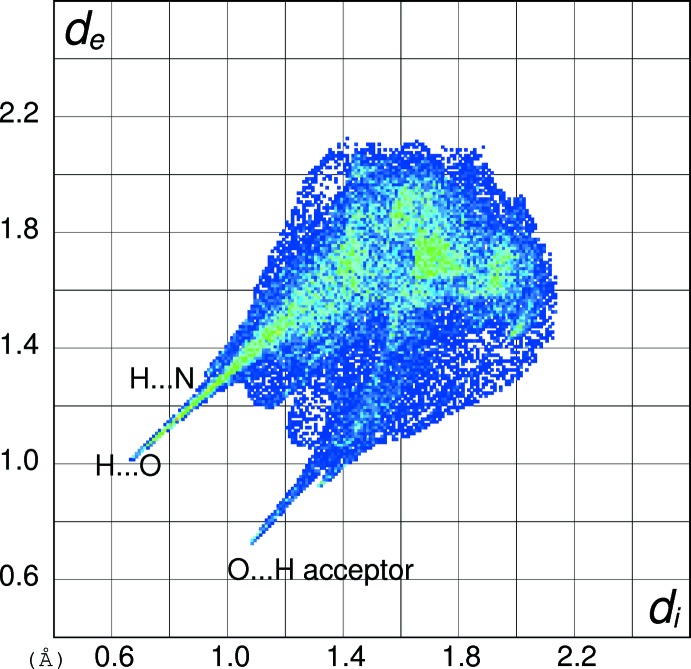
Hirshfeld fingerprint plot for the C_7_H_9_N_2_O_2_^+^ cation in (III).

**Figure 13:**
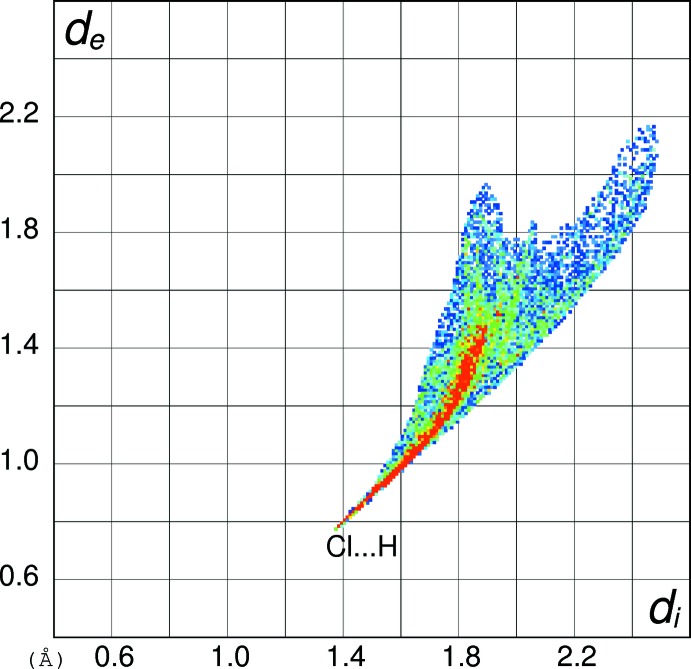
Hirshfeld fingerprint plot for the chloride anion in (I).

**Figure 14:**
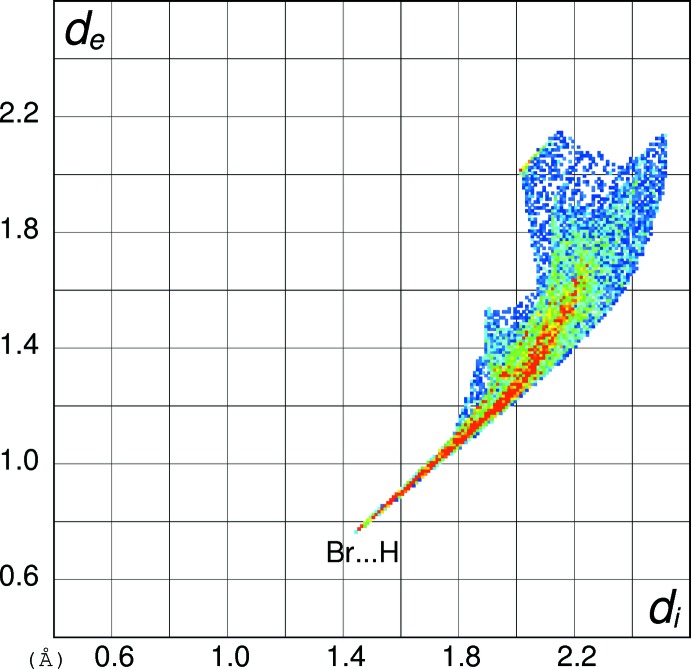
Hirshfeld fingerprint plot for the bromide anion in (II).

The fingerprint plot for the cation in (I) (Fig. 10 ▸) of outward (*i.e.* non-reciprocal) contacts shows three prominent features: the spike ending at (*d*_i_, *d*_e_) = (∼0.76, ∼1.36 Å) and extending backwards corresponds to the short inter­molecular H⋯Cl contacts associated with the N—H⋯Cl hydrogen bonds. The pronounced (0.65, 1.00 Å) feature equates with the H⋯O (donor) contact of the O—H⋯O hydrogen bond and that at (1.00, 0.65 Å) is associated with the H⋯O (acceptor) contact. The fingerprint plot for the cation in (II) (Fig. 11 ▸) shows the equivalent three spikes ending at (0.76, 1.45), (0.72, 1.08) and (1.06, 0.72 Å): the greater value of *d*_e_ for the first of these presumably reflects the larger size of the bromide ion in (II) compared to the chloride ion in (I). The fingerprint plot for the cation in (III) (Fig. 12 ▸) naturally lacks the H⋯*X* (*X* = Cl, Br) features and has a more symmetric appearance, with the spike at (0.68, 1.02) equating to H⋯O (donor) and that at (1.08, 0.74 Å) equating to the O⋯H (acceptor) contact. The H⋯N (donor) contact is just perceptible as a shoulder-like feature terminating at (0.94, 1.30 Å) but mostly superimposed on the tail of the H⋯O spike. The ‘wing’ like fingerprint plot for the chloride ion in (I) (Fig. 13 ▸) looks radically different to that of the cation (Fig. 10 ▸) although the end-point at (1.38, 0.77 Å) of the sweeping feature corresponds well with the H⋯Cl contact for the cation. The fingerprint plot for the bromide ion in (II) (Fig. 14 ▸) with its sweeping feature terminating at (1.45, 0.77 Å) shows similar correspondence with the H⋯Br spike for the cation in (II) (Fig. 11 ▸).

## Database survey   

**Figure 15:**
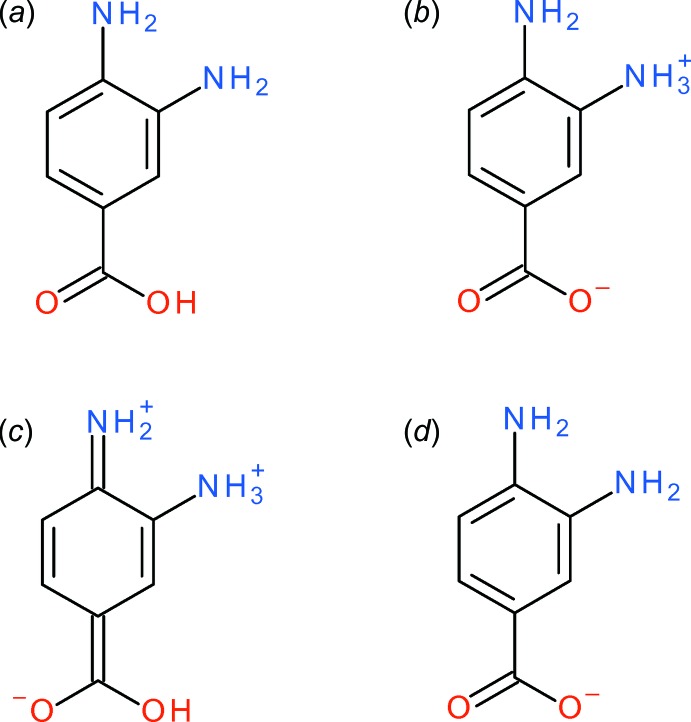
Different structures based on 3,4-di­amino­benzoic acid: (*a*) neutral C_7_H_8_N_2_O_2_ mol­ecule as found in DONDUH; (*b*) zwitterion in VODWIU; (*c*) quinoid resonance form of the C_7_H_9_N_2_O_2_^+^ cation in the title compounds (see text and compare scheme 1); (*d*) C_7_H_7_N_2_O_2_^−^ anion as found in the metal complexes noted in the text.

So far as we are aware, no mol­ecular salts containing the C_7_H_9_N_2_O_2_^+^ cation have been structurally characterized up to this point. A search of the Cambridge Structural Database (Version 5.40, updated to February 2020; Groom *et al.*, 2016 ▸) yielded five complexes of the 3,4-di­amino­benzoate *anion* (*i.e.* C_7_H_7_N_2_O_2_^−^) with sodium (CCDC refcode BEHJEE; Rzaczyńska *et al.*, 2003 ▸), zinc (MIWSES; Fernández-Palacio *et al.*, 2014 ▸), tin (XUPRUV and XUPSAC; Pruchnik *et al.*, 2002 ▸) and neodymium (YENKOR; Rzaczyńska *et al.*, 1994 ▸). As noted above, the structure of the zwitterionic free mol­ecule of C_7_H_8_N_2_O_2_ is known (VODWIU; Rzaczyńska *et al.*, 2000 ▸). Inter­estingly, the neutral, non-zwitterionic form of C_7_H_8_N_2_O_2_ has been co-crystallized with an organo-rhenium compound and other species (DONDUH; Davies *et al.*, 2014 ▸). In VODWIU, the –CO_2_^−^ group accepts several N—H⋯O hydrogen bonds while in DONDUH pairwise carb­oxy­lic-acid inversion dimers are formed. The different possible structures of the organic species are shown in Fig. 15 ▸.

## Synthesis and crystallization   

Equimolar mixtures of 3,4-di­amino­benzoic acid and hydro­chloric acid (I), hydro­bromic acid (II) and nitric acid (III) dissolved in water were deca­nted into petri dishes at room temperature and brown plates of (I), pale-brown laths of (II) and colourless slabs of (III) formed as the water evaporated over the course of a few days. The IR spectra for 3,4-di­amino­benzoic acid, (I), (II) and (III) are available as supporting information.

## Refinement   

Crystal data, data collection and structure refinement details are summarized in Table 6 ▸. For each structure, the N-bound H atoms were located in difference maps and their positions were freely refined. The C-bound H atoms were geometrically placed (C—H = 0.95 Å) and refined as riding atoms. The water H atoms in (III) were located in difference maps and refined as riding atoms in their as-found relative locations. The constraint *U*_iso_(H) = 1.2*U*_eq_(carrier) was applied in all cases. The crystal used for the data collection of (II) was twinned by rotation about [100] in reciprocal space in a 0.4896 (15):0.5104 (15) ratio and data merging was not performed (*i.e.* HKLF 5 refinement). The absolute structure of (III) could not be determined based on the refinement reported here.

## Supplementary Material

Crystal structure: contains datablock(s) I, II, III, global. DOI: 10.1107/S2056989020003163/xi2026sup1.cif

Structure factors: contains datablock(s) I. DOI: 10.1107/S2056989020003163/xi2026Isup2.hkl

Structure factors: contains datablock(s) II. DOI: 10.1107/S2056989020003163/xi2026IIsup3.hkl

Structure factors: contains datablock(s) III. DOI: 10.1107/S2056989020003163/xi2026IIIsup4.hkl

Supporting information file. DOI: 10.1107/S2056989020003163/xi2026Isup5.cml

Supporting information file. DOI: 10.1107/S2056989020003163/xi2026IIsup6.cml

Supporting information file. DOI: 10.1107/S2056989020003163/xi2026IIIsup7.cml

CCDC references: 1988618, 1988617, 1988616

Additional supporting information:  crystallographic information; 3D view; checkCIF report

## Figures and Tables

**Table 1:** Hydrogen-bond geometry (Å, °) for (I)

*D*—H⋯*A*	*D*—H	H⋯*A*	*D*⋯*A*	*D*—H⋯*A*
O1—H1*O*⋯O2^i^	0.74 (3)	1.88 (3)	2.6169 (18)	170 (3)
N1—H1*N*⋯Cl1^ii^	0.90 (2)	2.32 (2)	3.1578 (14)	153.4 (17)
N1—H2*N*⋯Cl1	0.91 (2)	2.25 (2)	3.1576 (15)	172.1 (18)
N1—H3*N*⋯Cl1^iii^	0.90 (2)	2.49 (2)	3.1722 (14)	132.9 (16)
N2—H4*N*⋯Cl1^iv^	0.86 (2)	2.72 (2)	3.2382 (15)	120.3 (17)
N2—H5*N*⋯Cl1^iii^	0.81 (2)	2.68 (2)	3.4852 (15)	176 (2)

Symmetry codes: (i) 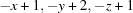; (ii) 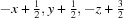; (iii) 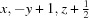; (iv) 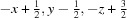.

**Table 2:** Hydrogen-bond geometry (Å, °) for (II)

*D*—H⋯*A*	*D*—H	H⋯*A*	*D*⋯*A*	*D*—H⋯*A*
O1—H1*O*⋯Br1^i^	0.79 (7)	2.42 (7)	3.199 (3)	169 (6)
N1—H1*N*⋯Br1	0.73 (6)	2.70 (7)	3.404 (5)	163 (6)
N1—H2*N*⋯O2^ii^	0.90 (6)	1.90 (6)	2.787 (5)	168 (5)
N1—H3*N*⋯Br1^iii^	0.85 (6)	2.49 (6)	3.333 (5)	171 (5)
N2—H4*N*⋯Br1^iv^	0.81 (7)	2.98 (7)	3.705 (5)	150 (5)
N2—H5*N*⋯Br1^v^	0.82 (6)	2.98 (6)	3.513 (4)	125 (5)
C2—H2⋯O2^ii^	0.95	2.23	3.024 (5)	140

Symmetry codes: (i) 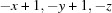; (ii) 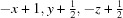; (iii) 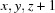; (iv) 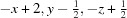; (v) 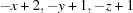.

**Table 3:** Hydrogen-bond geometry (Å, °) for (III)

*D*—H⋯*A*	*D*—H	H⋯*A*	*D*⋯*A*	*D*—H⋯*A*
N1—H1*N*⋯O6^i^	0.91	1.87	2.764 (5)	165
N1—H2*N*⋯O2^ii^	0.91	1.91	2.808 (5)	168
N1—H3*N*⋯N2^iii^	0.91	2.32	3.094 (6)	143
N1—H3*N*⋯O6^iv^	0.91	2.33	2.862 (5)	118
N2—H4*N*⋯O4^i^	0.91 (6)	2.07 (6)	2.972 (5)	169 (5)
N2—H5*N*⋯O5^v^	0.83 (6)	2.34 (7)	3.129 (5)	159 (6)
O1—H1*O*⋯O3	0.88 (6)	1.79 (6)	2.662 (5)	170 (6)
O6—H2*O*⋯O5	0.86	2.02	2.884 (5)	176
O6—H3*O*⋯O3^vi^	0.96	2.26	2.920 (5)	125
O6—H3*O*⋯O4^vi^	0.96	2.33	3.068 (5)	133
C2—H2⋯O2^ii^	0.95	2.54	3.285 (5)	135

Symmetry codes: (i) 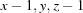; (ii) 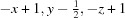; (iii) 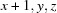; (iv) 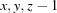; (v) 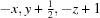; (vi) 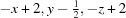.

**Table 4:** Aromatic π–π stacking inter­actions in the title compounds All inter­actions involve the C1–C6 benzene rings. *Cg*⋯*Cg* is the centroid–centroid separation, α is the dihedral angle between the ring planes.

Compound	*Cg*⋯*Cg* (Å)	α (°)	slippage (Å)	symmetry
(I)	3.8895 (9)	1.79 (7)	1.902	*x*, 1 − *y*, *z* − 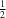
(I)	3.8895 (9)	1.79 (7)	1.822	*x*, 1 − *y*, *z* + 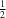
(II)	3.736 (3)	1.5 (2)	2.035	*x*, 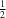 − *y*, *z* − 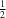
(II)	3.736 (3)	1.5 (2)	1.954	*x*, 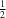 − *y*, *z* + 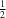
(III)	3.890 (2)	0.0 (2)	2.105	*x* − 1, *y*, *z*
(III)	3.890 (2)	0.0 (2)	2.105	*x* + 1, *y*, *z*

**Table 5:** Hirshfeld fingerprint contact percentages for different inter­molecular inter­actions in the title compounds

Inter­action	(I)	(II)	(III)
H⋯H	30.3	30.7	23.3
H⋯*X* ^*a*^	14.5	15.2	–
H⋯O (donor)	10.6	10.0	32.2
H⋯C	6.8	6.7	5.8
H⋯N	1.9	2.2	2.3
C⋯C	6.7	6.8	6.4
C⋯H	8.6	8.6	9.0
C⋯O	2.2	2.4	2.9
N⋯H	2.1	2.7	2.1
O⋯H (acceptor)	13.3	11.6	12.6
*X* ^*a*^⋯H	99.8	98.7	–

Note: (*a*) For (I), *X* = Cl; for (II) *X* = Br.

**Table 6:** Experimental details

	(I)	(II)	(III)
Crystal data			
Chemical formula	C_7_H_9_N_2_O_2_^+^·Cl^−^	C_7_H_9_N_2_O_2_^+^·Br^−^	C_7_H_9_N_2_O_2_^+^·NO_3_^−^·H_2_O
*M*_r_	188.61	233.07	233.19
Crystal system, space group	Monoclinic, *C*2/*c*	Monoclinic, *P*2_1_/*c*	Monoclinic, *P*2_1_
Temperature (K)	100	100	100
*a*, *b*, *c* (Å)	28.0521 (6), 8.0469 (2), 7.2511 (2)	12.1190 (7), 11.0708 (5), 6.3795 (3)	3.8899 (3), 9.8238 (6), 12.5799 (9)
β (°)	91.309 (2)	103.047 (6)	98.673 (7)
*V* (Å^3^)	1636.38 (7)	833.82 (8)	475.22 (6)
*Z*	8	4	2
Radiation type	Mo *K*α	Mo *K*α	Mo *K*α
μ (mm^−1^)	0.43	4.89	0.14
Crystal size (mm)	0.41 × 0.26 × 0.04	0.25 × 0.12 × 0.04	0.20 × 0.10 × 0.05

Data collection			
Diffractometer	Rigaku Mercury CCD	Rigaku Mercury CCD	Rigaku Mercury CCD
Absorption correction	Gaussian (*CrysAlis PRO*; Rigaku, 2017 ▸)	Multi-scan (*CrysAlis PRO*; Rigaku, 2017 ▸)	Multi-scan (*CrysAlis PRO*; Rigaku, 2017 ▸)
*T*_min_, *T*_max_	0.624, 1.000	0.538, 1.000	0.861, 1.000
No. of measured, independent and observed [*I* > 2σ(*I*)] reflections	13456, 1865, 1765	3343, 3343, 3031	10770, 2157, 2100
*R*_int_	0.035	?	0.067
(sin θ/λ)_max_ (Å^−1^)	0.649	0.649	0.649

Refinement			
*R*[*F* ^2^ > 2σ(*F* ^2^)], *wR*(*F* ^2^), *S*	0.031, 0.093, 1.13	0.036, 0.100, 1.14	0.058, 0.149, 1.15
No. of reflections	1865	3343	2157
No. of parameters	127	128	155
No. of restraints	0	0	1
H-atom treatment	H atoms treated by a mixture of independent and constrained refinement	H atoms treated by a mixture of independent and constrained refinement	H atoms treated by a mixture of independent and constrained refinement
Δρ_max_, Δρ_min_ (e Å^−3^)	0.45, −0.27	1.01, −0.70	0.48, −0.37

Computer programs: *CrysAlis PRO* (Rigaku, 2017 ▸), *SHELXS97* (Sheldrick, 2008 ▸), *SHELXL2014/7* (Sheldrick, 2015 ▸), *ORTEP-3 for Windows* (Farrugia, 2012 ▸) and *publCIF* (Westrip, 2010 ▸).

## supplementary crystallographic information

### 2-Amino-5-carboxyanilinium chloride (I) Crystal data

**Table:**

C_7_H_9_N_2_O_2_^+^·Cl^−^	*F*(000) = 784
*M**_r_* = 188.61	*D*_x_ = 1.531 Mg m^−^^3^
Monoclinic, *C*2/*c*	Mo *K*α radiation, λ = 0.71073 Å
*a* = 28.0521 (6) Å	Cell parameters from 8736 reflections
*b* = 8.0469 (2) Å	θ = 2.9–30.1°
*c* = 7.2511 (2) Å	µ = 0.43 mm^−^^1^
β = 91.309 (2)°	*T* = 100 K
*V* = 1636.38 (7) Å^3^	Plate, brown
*Z* = 8	0.41 × 0.26 × 0.04 mm

### 2-Amino-5-carboxyanilinium chloride (I) Data collection

**Table:**

Rigaku Mercury CCD diffractometer	1765 reflections with *I* > 2σ(*I*)
ω scans	*R*_int_ = 0.035
Absorption correction: gaussian (CrysAlisPRO; Rigaku, 2017)	θ_max_ = 27.5°, θ_min_ = 2.6°
*T*_min_ = 0.624, *T*_max_ = 1.000	*h* = −36→36
13456 measured reflections	*k* = −10→10
1865 independent reflections	*l* = −9→9

### 2-Amino-5-carboxyanilinium chloride (I) Refinement

**Table:**

Refinement on *F*^2^	Primary atom site location: structure-invariant direct methods
Least-squares matrix: full	Hydrogen site location: mixed
*R*[*F*^2^ > 2σ(*F*^2^)] = 0.031	H atoms treated by a mixture of independent and constrained refinement
*wR*(*F*^2^) = 0.093	*w* = 1/[σ^2^(*F*_o_^2^) + (0.0434*P*)^2^ + 2.8134*P*] where *P* = (*F*_o_^2^ + 2*F*_c_^2^)/3
*S* = 1.13	(Δ/σ)_max_ = 0.002
1865 reflections	Δρ_max_ = 0.45 e Å^−^^3^
127 parameters	Δρ_min_ = −0.27 e Å^−^^3^
0 restraints	

### 2-Amino-5-carboxyanilinium chloride (I) Special details

**Table:**

Geometry. All esds (except the esd in the dihedral angle between two l.s. planes) are estimated using the full covariance matrix. The cell esds are taken into account individually in the estimation of esds in distances, angles and torsion angles; correlations between esds in cell parameters are only used when they are defined by crystal symmetry. An approximate (isotropic) treatment of cell esds is used for estimating esds involving l.s. planes.

### 2-Amino-5-carboxyanilinium chloride (I) Fractional atomic coordinates and isotropic or equivalent isotropic displacement parameters (Å^2^)

**Table:**

	*x*	*y*	*z*	*U*_iso_*/*U*_eq_	
C1	0.42198 (5)	0.7087 (2)	0.6424 (2)	0.0132 (3)	
C2	0.37721 (5)	0.7561 (2)	0.7037 (2)	0.0124 (3)	
H2	0.3696	0.8704	0.7173	0.015*	
C3	0.34405 (5)	0.6353 (2)	0.7443 (2)	0.0115 (3)	
C4	0.35376 (5)	0.46541 (19)	0.7258 (2)	0.0118 (3)	
C5	0.39962 (5)	0.4200 (2)	0.6688 (2)	0.0132 (3)	
H5	0.4077	0.3058	0.6586	0.016*	
C6	0.43292 (6)	0.5395 (2)	0.6276 (2)	0.0139 (3)	
H6	0.4636	0.5067	0.5887	0.017*	
C7	0.45674 (6)	0.8354 (2)	0.5858 (2)	0.0150 (3)	
N1	0.29675 (5)	0.68573 (18)	0.8047 (2)	0.0126 (3)	
H1N	0.2945 (7)	0.797 (3)	0.818 (3)	0.015*	
H2N	0.2748 (7)	0.653 (3)	0.717 (3)	0.015*	
H3N	0.2895 (7)	0.641 (3)	0.914 (3)	0.015*	
N2	0.31909 (5)	0.34702 (18)	0.7525 (2)	0.0158 (3)	
H4N	0.3282 (7)	0.246 (3)	0.768 (3)	0.019*	
H5N	0.2970 (8)	0.371 (3)	0.817 (3)	0.019*	
O1	0.44480 (5)	0.98914 (17)	0.6117 (2)	0.0226 (3)	
H1O	0.4634 (9)	1.042 (3)	0.570 (3)	0.027*	
O2	0.49517 (4)	0.79238 (15)	0.51547 (18)	0.0203 (3)	
Cl1	0.22232 (2)	0.53660 (4)	0.51641 (5)	0.01266 (14)	

### 2-Amino-5-carboxyanilinium chloride (I) Atomic displacement parameters (Å^2^)

**Table:**

	*U*^11^	*U*^22^	*U*^33^	*U*^12^	*U*^13^	*U*^23^
C1	0.0110 (7)	0.0134 (8)	0.0153 (7)	−0.0017 (6)	0.0015 (5)	0.0010 (6)
C2	0.0130 (7)	0.0109 (7)	0.0132 (7)	−0.0002 (6)	0.0004 (5)	0.0004 (6)
C3	0.0107 (7)	0.0131 (7)	0.0108 (7)	0.0007 (6)	0.0011 (5)	−0.0002 (6)
C4	0.0128 (7)	0.0123 (8)	0.0104 (7)	−0.0010 (5)	−0.0002 (5)	0.0013 (5)
C5	0.0144 (7)	0.0107 (7)	0.0145 (7)	0.0019 (6)	0.0011 (5)	0.0001 (6)
C6	0.0111 (7)	0.0150 (8)	0.0157 (8)	0.0012 (6)	0.0019 (6)	0.0003 (6)
C7	0.0129 (7)	0.0141 (8)	0.0180 (8)	−0.0013 (6)	0.0015 (6)	0.0014 (6)
N1	0.0124 (6)	0.0106 (7)	0.0149 (7)	0.0001 (5)	0.0032 (5)	0.0008 (5)
N2	0.0144 (7)	0.0094 (7)	0.0236 (7)	0.0003 (5)	0.0050 (5)	0.0014 (6)
O1	0.0181 (6)	0.0132 (6)	0.0370 (8)	−0.0040 (5)	0.0096 (5)	0.0020 (5)
O2	0.0130 (5)	0.0189 (6)	0.0294 (7)	−0.0015 (5)	0.0074 (5)	0.0026 (5)
Cl1	0.0148 (2)	0.0094 (2)	0.0138 (2)	0.00125 (12)	0.00239 (14)	0.00038 (12)

### 2-Amino-5-carboxyanilinium chloride (I) Geometric parameters (Å, º)

**Table:**

C1—C2	1.395 (2)	C5—H5	0.9500
C1—C6	1.401 (2)	C6—H6	0.9500
C1—C7	1.475 (2)	C7—O2	1.252 (2)
C2—C3	1.382 (2)	C7—O1	1.296 (2)
C2—H2	0.9500	N1—H1N	0.90 (2)
C3—C4	1.401 (2)	N1—H2N	0.91 (2)
C3—N1	1.4640 (19)	N1—H3N	0.90 (2)
C4—N2	1.378 (2)	N2—H4N	0.86 (2)
C4—C5	1.408 (2)	N2—H5N	0.81 (2)
C5—C6	1.378 (2)	O1—H1O	0.74 (3)
			
C2—C1—C6	119.38 (14)	C5—C6—H6	119.6
C2—C1—C7	120.35 (15)	C1—C6—H6	119.6
C6—C1—C7	120.22 (14)	O2—C7—O1	123.41 (15)
C3—C2—C1	119.42 (15)	O2—C7—C1	120.19 (15)
C3—C2—H2	120.3	O1—C7—C1	116.40 (14)
C1—C2—H2	120.3	C3—N1—H1N	111.9 (13)
C2—C3—C4	122.18 (14)	C3—N1—H2N	108.2 (12)
C2—C3—N1	119.22 (14)	H1N—N1—H2N	108.3 (18)
C4—C3—N1	118.59 (14)	C3—N1—H3N	112.1 (13)
N2—C4—C3	121.45 (14)	H1N—N1—H3N	106.6 (19)
N2—C4—C5	120.93 (15)	H2N—N1—H3N	109.6 (18)
C3—C4—C5	117.53 (14)	C4—N2—H4N	117.6 (14)
C6—C5—C4	120.72 (15)	C4—N2—H5N	118.0 (16)
C6—C5—H5	119.6	H4N—N2—H5N	112 (2)
C4—C5—H5	119.6	C7—O1—H1O	107.7 (19)
C5—C6—C1	120.73 (14)		
			
C6—C1—C2—C3	−1.6 (2)	C3—C4—C5—C6	−2.0 (2)
C7—C1—C2—C3	175.73 (14)	C4—C5—C6—C1	0.3 (2)
C1—C2—C3—C4	−0.1 (2)	C2—C1—C6—C5	1.5 (2)
C1—C2—C3—N1	−178.67 (14)	C7—C1—C6—C5	−175.82 (15)
C2—C3—C4—N2	−174.64 (15)	C2—C1—C7—O2	−173.60 (15)
N1—C3—C4—N2	3.9 (2)	C6—C1—C7—O2	3.7 (2)
C2—C3—C4—C5	1.9 (2)	C2—C1—C7—O1	5.8 (2)
N1—C3—C4—C5	−179.57 (13)	C6—C1—C7—O1	−176.96 (16)
N2—C4—C5—C6	174.58 (15)		

### 2-Amino-5-carboxyanilinium chloride (I) Hydrogen-bond geometry (Å, º)

**Table:**

*D*—H···*A*	*D*—H	H···*A*	*D*···*A*	*D*—H···*A*
O1—H1*O*···O2^i^	0.74 (3)	1.88 (3)	2.6169 (18)	170 (3)
N1—H1*N*···Cl1^ii^	0.90 (2)	2.32 (2)	3.1578 (14)	153.4 (17)
N1—H2*N*···Cl1	0.91 (2)	2.25 (2)	3.1576 (15)	172.1 (18)
N1—H3*N*···Cl1^iii^	0.90 (2)	2.49 (2)	3.1722 (14)	132.9 (16)
N2—H4*N*···Cl1^iv^	0.86 (2)	2.72 (2)	3.2382 (15)	120.3 (17)
N2—H5*N*···Cl1^iii^	0.81 (2)	2.68 (2)	3.4852 (15)	176 (2)

Symmetry codes: (i) −*x*+1, −*y*+2, −*z*+1; (ii) −*x*+1/2, *y*+1/2, −*z*+3/2; (iii) *x*, −*y*+1, *z*+1/2; (iv) −*x*+1/2, *y*−1/2, −*z*+3/2.

### 2-Amino-5-carboxyanilinium bromide (II) Crystal data

**Table:**

C_7_H_9_N_2_O_2_^+^·Br^−^	*F*(000) = 464
*M**_r_* = 233.07	*D*_x_ = 1.857 Mg m^−^^3^
Monoclinic, *P*2_1_/*c*	Mo *K*α radiation, λ = 0.71073 Å
*a* = 12.1190 (7) Å	Cell parameters from 8521 reflections
*b* = 11.0708 (5) Å	θ = 3.4–30.1°
*c* = 6.3795 (3) Å	µ = 4.89 mm^−^^1^
β = 103.047 (6)°	*T* = 100 K
*V* = 833.82 (8) Å^3^	Lath, pale brown
*Z* = 4	0.25 × 0.12 × 0.04 mm

### 2-Amino-5-carboxyanilinium bromide (II) Data collection

**Table:**

Rigaku Mercury CCD diffractometer	3031 reflections with *I* > 2σ(*I*)
ω scans	θ_max_ = 27.5°, θ_min_ = 2.5°
Absorption correction: multi-scan (CrysAlisPRO; Rigaku, 2017)	*h* = −15→15
*T*_min_ = 0.538, *T*_max_ = 1.000	*k* = −14→14
3343 measured reflections	*l* = −8→8
3343 independent reflections	

### 2-Amino-5-carboxyanilinium bromide (II) Refinement

**Table:**

Refinement on *F*^2^	Primary atom site location: structure-invariant direct methods
Least-squares matrix: full	Hydrogen site location: mixed
*R*[*F*^2^ > 2σ(*F*^2^)] = 0.036	H atoms treated by a mixture of independent and constrained refinement
*wR*(*F*^2^) = 0.100	*w* = 1/[σ^2^(*F*_o_^2^) + (0.0423*P*)^2^ + 2.9863*P*] where *P* = (*F*_o_^2^ + 2*F*_c_^2^)/3
*S* = 1.14	(Δ/σ)_max_ = 0.001
3343 reflections	Δρ_max_ = 1.01 e Å^−^^3^
128 parameters	Δρ_min_ = −0.69 e Å^−^^3^
0 restraints	

### 2-Amino-5-carboxyanilinium bromide (II) Special details

**Table:**

Geometry. All esds (except the esd in the dihedral angle between two l.s. planes) are estimated using the full covariance matrix. The cell esds are taken into account individually in the estimation of esds in distances, angles and torsion angles; correlations between esds in cell parameters are only used when they are defined by crystal symmetry. An approximate (isotropic) treatment of cell esds is used for estimating esds involving l.s. planes.
Refinement. Refined as a two-component twin

### 2-Amino-5-carboxyanilinium bromide (II) Fractional atomic coordinates and isotropic or equivalent isotropic displacement parameters (Å^2^)

**Table:**

	*x*	*y*	*z*	*U*_iso_*/*U*_eq_	
C1	0.5791 (4)	0.3232 (4)	0.3020 (7)	0.0114 (9)	
C2	0.6253 (4)	0.4394 (4)	0.3348 (7)	0.0105 (8)	
H2	0.5781	0.5085	0.3035	0.013*	
C3	0.7405 (4)	0.4520 (4)	0.4131 (7)	0.0093 (8)	
C4	0.8130 (4)	0.3529 (4)	0.4643 (7)	0.0110 (9)	
C5	0.7646 (4)	0.2369 (4)	0.4251 (7)	0.0113 (9)	
H5	0.8119	0.1677	0.4535	0.014*	
C6	0.6494 (4)	0.2226 (4)	0.3460 (7)	0.0122 (9)	
H6	0.6181	0.1438	0.3216	0.015*	
C7	0.4558 (4)	0.3082 (4)	0.2165 (7)	0.0107 (9)	
N1	0.7876 (4)	0.5748 (3)	0.4373 (8)	0.0110 (8)	
H1N	0.816 (5)	0.593 (5)	0.352 (10)	0.013*	
H2N	0.729 (5)	0.627 (5)	0.409 (9)	0.013*	
H3N	0.815 (5)	0.590 (5)	0.569 (10)	0.013*	
N2	0.9285 (4)	0.3686 (4)	0.5425 (7)	0.0155 (9)	
H4N	0.959 (5)	0.304 (6)	0.578 (9)	0.019*	
H5N	0.951 (5)	0.420 (6)	0.635 (10)	0.019*	
O1	0.3992 (3)	0.4115 (3)	0.1817 (6)	0.0167 (7)	
H1O	0.334 (6)	0.400 (5)	0.130 (10)	0.020*	
O2	0.4107 (3)	0.2098 (3)	0.1823 (6)	0.0161 (7)	
Br1	0.86980 (4)	0.61815 (4)	−0.03325 (7)	0.01281 (15)	

### 2-Amino-5-carboxyanilinium bromide (II) Atomic displacement parameters (Å^2^)

**Table:**

	*U*^11^	*U*^22^	*U*^33^	*U*^12^	*U*^13^	*U*^23^
C1	0.011 (2)	0.009 (2)	0.013 (2)	−0.0010 (16)	0.0028 (17)	0.0006 (16)
C2	0.013 (2)	0.009 (2)	0.010 (2)	0.0014 (16)	0.0034 (16)	−0.0008 (16)
C3	0.014 (2)	0.005 (2)	0.010 (2)	−0.0009 (16)	0.0047 (17)	−0.0012 (16)
C4	0.013 (2)	0.012 (2)	0.008 (2)	0.0008 (17)	0.0021 (16)	0.0011 (16)
C5	0.014 (2)	0.011 (2)	0.010 (2)	0.0028 (17)	0.0048 (17)	0.0011 (17)
C6	0.016 (2)	0.009 (2)	0.012 (2)	−0.0017 (16)	0.0033 (18)	−0.0015 (17)
C7	0.010 (2)	0.011 (2)	0.012 (2)	0.0009 (16)	0.0032 (17)	0.0000 (17)
N1	0.0112 (19)	0.0086 (18)	0.013 (2)	−0.0019 (15)	0.0034 (16)	−0.0003 (16)
N2	0.010 (2)	0.015 (2)	0.020 (2)	0.0019 (17)	0.0002 (16)	0.0013 (17)
O1	0.0083 (16)	0.0094 (15)	0.029 (2)	0.0007 (12)	−0.0025 (14)	0.0008 (13)
O2	0.0118 (15)	0.0097 (15)	0.0247 (19)	−0.0020 (13)	−0.0001 (14)	−0.0012 (13)
Br1	0.0108 (2)	0.0136 (2)	0.0130 (2)	−0.00047 (18)	0.00053 (16)	−0.00011 (17)

### 2-Amino-5-carboxyanilinium bromide (II) Geometric parameters (Å, º)

**Table:**

C1—C6	1.391 (6)	C5—H5	0.9500
C1—C2	1.400 (6)	C6—H6	0.9500
C1—C7	1.479 (6)	C7—O2	1.216 (5)
C2—C3	1.379 (6)	C7—O1	1.326 (5)
C2—H2	0.9500	N1—H1N	0.73 (6)
C3—C4	1.398 (6)	N1—H2N	0.90 (6)
C3—N1	1.468 (5)	N1—H3N	0.85 (6)
C4—N2	1.387 (6)	N2—H4N	0.81 (7)
C4—C5	1.410 (6)	N2—H5N	0.82 (6)
C5—C6	1.383 (6)	O1—H1O	0.79 (7)
			
C6—C1—C2	119.9 (4)	C5—C6—H6	119.9
C6—C1—C7	120.4 (4)	C1—C6—H6	119.9
C2—C1—C7	119.7 (4)	O2—C7—O1	123.2 (4)
C3—C2—C1	119.1 (4)	O2—C7—C1	122.8 (4)
C3—C2—H2	120.5	O1—C7—C1	114.0 (4)
C1—C2—H2	120.5	C3—N1—H1N	114 (5)
C2—C3—C4	122.4 (4)	C3—N1—H2N	107 (4)
C2—C3—N1	118.1 (4)	H1N—N1—H2N	99 (6)
C4—C3—N1	119.5 (4)	C3—N1—H3N	111 (4)
N2—C4—C3	121.1 (4)	H1N—N1—H3N	122 (6)
N2—C4—C5	121.6 (4)	H2N—N1—H3N	101 (5)
C3—C4—C5	117.3 (4)	C4—N2—H4N	110 (4)
C6—C5—C4	120.9 (4)	C4—N2—H5N	119 (5)
C6—C5—H5	119.5	H4N—N2—H5N	111 (6)
C4—C5—H5	119.5	C7—O1—H1O	111 (4)
C5—C6—C1	120.3 (4)		
			
C6—C1—C2—C3	−0.7 (6)	C3—C4—C5—C6	−2.1 (7)
C7—C1—C2—C3	−179.5 (4)	C4—C5—C6—C1	0.5 (7)
C1—C2—C3—C4	−1.0 (7)	C2—C1—C6—C5	1.0 (7)
C1—C2—C3—N1	177.4 (4)	C7—C1—C6—C5	179.8 (4)
C2—C3—C4—N2	179.9 (4)	C6—C1—C7—O2	0.4 (7)
N1—C3—C4—N2	1.5 (7)	C2—C1—C7—O2	179.2 (4)
C2—C3—C4—C5	2.4 (7)	C6—C1—C7—O1	−179.9 (4)
N1—C3—C4—C5	−176.0 (4)	C2—C1—C7—O1	−1.1 (6)
N2—C4—C5—C6	−179.6 (5)		

### 2-Amino-5-carboxyanilinium bromide (II) Hydrogen-bond geometry (Å, º)

**Table:**

*D*—H···*A*	*D*—H	H···*A*	*D*···*A*	*D*—H···*A*
O1—H1*O*···Br1^i^	0.79 (7)	2.42 (7)	3.199 (3)	169 (6)
N1—H1*N*···Br1	0.73 (6)	2.70 (7)	3.404 (5)	163 (6)
N1—H2*N*···O2^ii^	0.90 (6)	1.90 (6)	2.787 (5)	168 (5)
N1—H3*N*···Br1^iii^	0.85 (6)	2.49 (6)	3.333 (5)	171 (5)
N2—H4*N*···Br1^iv^	0.81 (7)	2.98 (7)	3.705 (5)	150 (5)
N2—H5*N*···Br1^v^	0.82 (6)	2.98 (6)	3.513 (4)	125 (5)
C2—H2···O2^ii^	0.95	2.23	3.024 (5)	140

Symmetry codes: (i) −*x*+1, −*y*+1, −*z*; (ii) −*x*+1, *y*+1/2, −*z*+1/2; (iii) *x*, *y*, *z*+1; (iv) −*x*+2, *y*−1/2, −*z*+1/2; (v) −*x*+2, −*y*+1, −*z*+1.

### 2-Amino-5-carboxyanilinium nitrate monohydrate (III) Crystal data

**Table:**

C_7_H_9_N_2_O_2_^+^·NO_3_^−^·H_2_O	*F*(000) = 244
*M**_r_* = 233.19	*D*_x_ = 1.630 Mg m^−^^3^
Monoclinic, *P*2_1_	Mo *K*α radiation, λ = 0.71073 Å
*a* = 3.8899 (3) Å	Cell parameters from 3653 reflections
*b* = 9.8238 (6) Å	θ = 2.7–30.8°
*c* = 12.5799 (9) Å	µ = 0.14 mm^−^^1^
β = 98.673 (7)°	*T* = 100 K
*V* = 475.22 (6) Å^3^	Slab, colourless
*Z* = 2	0.20 × 0.10 × 0.05 mm

### 2-Amino-5-carboxyanilinium nitrate monohydrate (III) Data collection

**Table:**

Rigaku Mercury CCD diffractometer	2100 reflections with *I* > 2σ(*I*)
ω scans	*R*_int_ = 0.067
Absorption correction: multi-scan (CrysAlisPRO; Rigaku, 2017)	θ_max_ = 27.5°, θ_min_ = 2.6°
*T*_min_ = 0.861, *T*_max_ = 1.000	*h* = −5→5
10770 measured reflections	*k* = −12→12
2157 independent reflections	*l* = −16→16

### 2-Amino-5-carboxyanilinium nitrate monohydrate (III) Refinement

**Table:**

Refinement on *F*^2^	Primary atom site location: structure-invariant direct methods
Least-squares matrix: full	Hydrogen site location: mixed
*R*[*F*^2^ > 2σ(*F*^2^)] = 0.058	H atoms treated by a mixture of independent and constrained refinement
*wR*(*F*^2^) = 0.149	*w* = 1/[σ^2^(*F*_o_^2^) + (0.0747*P*)^2^ + 0.3934*P*] where *P* = (*F*_o_^2^ + 2*F*_c_^2^)/3
*S* = 1.15	(Δ/σ)_max_ = 0.005
2157 reflections	Δρ_max_ = 0.48 e Å^−^^3^
155 parameters	Δρ_min_ = −0.37 e Å^−^^3^
1 restraint	

### 2-Amino-5-carboxyanilinium nitrate monohydrate (III) Special details

**Table:**

Geometry. All esds (except the esd in the dihedral angle between two l.s. planes) are estimated using the full covariance matrix. The cell esds are taken into account individually in the estimation of esds in distances, angles and torsion angles; correlations between esds in cell parameters are only used when they are defined by crystal symmetry. An approximate (isotropic) treatment of cell esds is used for estimating esds involving l.s. planes.

### 2-Amino-5-carboxyanilinium nitrate monohydrate (III) Fractional atomic coordinates and isotropic or equivalent isotropic displacement parameters (Å^2^)

**Table:**

	*x*	*y*	*z*	*U*_iso_*/*U*_eq_	
C1	0.2205 (11)	0.5688 (5)	0.4736 (3)	0.0120 (8)	
C2	0.2777 (10)	0.4617 (5)	0.4062 (3)	0.0121 (8)	
H2	0.4231	0.3880	0.4334	0.014*	
C3	0.1240 (10)	0.4617 (4)	0.2997 (3)	0.0120 (8)	
C4	−0.0999 (11)	0.5677 (4)	0.2573 (3)	0.0124 (8)	
C5	−0.1462 (11)	0.6769 (4)	0.3255 (3)	0.0128 (8)	
H5	−0.2878	0.7516	0.2982	0.015*	
C6	0.0082 (11)	0.6778 (4)	0.4303 (3)	0.0121 (8)	
H6	−0.0277	0.7530	0.4748	0.014*	
C7	0.3867 (11)	0.5736 (4)	0.5864 (3)	0.0127 (8)	
N1	0.1979 (10)	0.3534 (4)	0.2274 (3)	0.0137 (8)	
H1N	−0.0055	0.3198	0.1919	0.016*	
H2N	0.3174	0.2856	0.2661	0.016*	
H3N	0.3279	0.3871	0.1790	0.016*	
N2	−0.2843 (11)	0.5597 (4)	0.1542 (3)	0.0167 (8)	
H4N	−0.198 (16)	0.524 (6)	0.097 (5)	0.020*	
H5N	−0.358 (16)	0.637 (6)	0.136 (4)	0.020*	
O1	0.5752 (9)	0.4650 (3)	0.6185 (2)	0.0182 (7)	
H1O	0.685 (15)	0.469 (7)	0.685 (5)	0.022*	
O2	0.3588 (8)	0.6720 (3)	0.6453 (2)	0.0156 (7)	
N3	0.9275 (9)	0.4263 (4)	0.8918 (3)	0.0147 (8)	
O3	0.9624 (10)	0.4968 (4)	0.8102 (3)	0.0234 (8)	
O4	1.0903 (8)	0.4593 (4)	0.9805 (2)	0.0210 (7)	
O5	0.7311 (10)	0.3252 (4)	0.8821 (3)	0.0263 (8)	
O6	0.6514 (8)	0.2329 (3)	1.0947 (3)	0.0181 (7)	
H2O	0.6684	0.2566	1.0297	0.022*	
H3O	0.6328	0.1351	1.0976	0.022*	

### 2-Amino-5-carboxyanilinium nitrate monohydrate (III) Atomic displacement parameters (Å^2^)

**Table:**

	*U*^11^	*U*^22^	*U*^33^	*U*^12^	*U*^13^	*U*^23^
C1	0.0129 (19)	0.0080 (18)	0.0155 (19)	−0.0052 (15)	0.0033 (14)	0.0015 (16)
C2	0.0105 (17)	0.0076 (18)	0.019 (2)	0.0003 (16)	0.0059 (14)	0.0019 (16)
C3	0.0133 (19)	0.0064 (18)	0.0172 (19)	−0.0016 (17)	0.0055 (14)	−0.0018 (16)
C4	0.015 (2)	0.0084 (19)	0.015 (2)	0.0014 (16)	0.0058 (15)	0.0003 (17)
C5	0.015 (2)	0.0044 (18)	0.020 (2)	0.0005 (16)	0.0043 (15)	0.0006 (17)
C6	0.0125 (19)	0.0046 (17)	0.021 (2)	−0.0010 (15)	0.0076 (15)	0.0000 (16)
C7	0.013 (2)	0.0098 (19)	0.015 (2)	−0.0043 (16)	0.0020 (15)	0.0000 (17)
N1	0.0137 (17)	0.0123 (18)	0.0148 (17)	0.0055 (14)	0.0014 (13)	−0.0014 (14)
N2	0.023 (2)	0.0115 (18)	0.0151 (18)	0.0049 (15)	0.0003 (14)	0.0008 (15)
O1	0.0250 (17)	0.0146 (16)	0.0134 (14)	0.0011 (14)	−0.0017 (12)	0.0004 (13)
O2	0.0183 (16)	0.0123 (15)	0.0159 (14)	−0.0029 (12)	0.0017 (11)	−0.0030 (13)
N3	0.0165 (17)	0.0137 (18)	0.0137 (17)	0.0016 (14)	0.0021 (13)	−0.0029 (14)
O3	0.0313 (19)	0.0206 (17)	0.0176 (16)	−0.0073 (14)	0.0010 (13)	0.0017 (14)
O4	0.0226 (16)	0.0222 (18)	0.0176 (15)	−0.0014 (14)	0.0012 (12)	−0.0029 (13)
O5	0.032 (2)	0.0202 (18)	0.0268 (19)	−0.0136 (16)	0.0056 (15)	−0.0046 (15)
O6	0.0214 (16)	0.0113 (14)	0.0223 (16)	0.0000 (13)	0.0061 (13)	−0.0008 (12)

### 2-Amino-5-carboxyanilinium nitrate monohydrate (III) Geometric parameters (Å, º)

**Table:**

C1—C2	1.391 (6)	C7—O1	1.323 (6)
C1—C6	1.410 (6)	N1—H1N	0.9100
C1—C7	1.469 (5)	N1—H2N	0.9100
C2—C3	1.382 (5)	N1—H3N	0.9100
C2—H2	0.9500	N2—H4N	0.91 (6)
C3—C4	1.409 (6)	N2—H5N	0.83 (6)
C3—N1	1.457 (5)	O1—H1O	0.88 (6)
C4—N2	1.386 (5)	N3—O4	1.240 (5)
C4—C5	1.402 (6)	N3—O5	1.247 (5)
C5—C6	1.363 (6)	N3—O3	1.262 (5)
C5—H5	0.9500	O6—H2O	0.8625
C6—H6	0.9500	O6—H3O	0.9644
C7—O2	1.232 (5)		
			
C2—C1—C6	118.5 (4)	O2—C7—O1	123.0 (4)
C2—C1—C7	121.7 (4)	O2—C7—C1	122.7 (4)
C6—C1—C7	119.7 (4)	O1—C7—C1	114.3 (4)
C3—C2—C1	120.3 (4)	C3—N1—H1N	109.5
C3—C2—H2	119.8	C3—N1—H2N	109.5
C1—C2—H2	119.8	H1N—N1—H2N	109.5
C2—C3—C4	121.3 (4)	C3—N1—H3N	109.5
C2—C3—N1	120.5 (4)	H1N—N1—H3N	109.5
C4—C3—N1	118.2 (4)	H2N—N1—H3N	109.5
N2—C4—C5	121.3 (4)	C4—N2—H4N	124 (4)
N2—C4—C3	121.0 (4)	C4—N2—H5N	108 (4)
C5—C4—C3	117.6 (4)	H4N—N2—H5N	107 (5)
C6—C5—C4	121.2 (4)	C7—O1—H1O	115 (4)
C6—C5—H5	119.4	O4—N3—O5	121.2 (4)
C4—C5—H5	119.4	O4—N3—O3	119.0 (4)
C5—C6—C1	121.0 (4)	O5—N3—O3	119.8 (4)
C5—C6—H6	119.5	H2O—O6—H3O	108.8
C1—C6—H6	119.5		
			
C6—C1—C2—C3	−1.1 (6)	C3—C4—C5—C6	−2.7 (6)
C7—C1—C2—C3	−178.4 (4)	C4—C5—C6—C1	0.1 (6)
C1—C2—C3—C4	−1.6 (6)	C2—C1—C6—C5	1.8 (6)
C1—C2—C3—N1	176.7 (4)	C7—C1—C6—C5	179.2 (4)
C2—C3—C4—N2	−172.5 (4)	C2—C1—C7—O2	174.8 (4)
N1—C3—C4—N2	9.2 (6)	C6—C1—C7—O2	−2.5 (6)
C2—C3—C4—C5	3.4 (6)	C2—C1—C7—O1	−3.7 (6)
N1—C3—C4—C5	−174.9 (4)	C6—C1—C7—O1	179.0 (4)
N2—C4—C5—C6	173.3 (4)		

### 2-Amino-5-carboxyanilinium nitrate monohydrate (III) Hydrogen-bond geometry (Å, º)

**Table:**

*D*—H···*A*	*D*—H	H···*A*	*D*···*A*	*D*—H···*A*
N1—H1*N*···O6^i^	0.91	1.87	2.764 (5)	165
N1—H2*N*···O2^ii^	0.91	1.91	2.808 (5)	168
N1—H3*N*···N2^iii^	0.91	2.32	3.094 (6)	143
N1—H3*N*···O6^iv^	0.91	2.33	2.862 (5)	118
N2—H4*N*···O4^i^	0.91 (6)	2.07 (6)	2.972 (5)	169 (5)
N2—H5*N*···O5^v^	0.83 (6)	2.34 (7)	3.129 (5)	159 (6)
O1—H1*O*···O3	0.88 (6)	1.79 (6)	2.662 (5)	170 (6)
O6—H2*O*···O5	0.86	2.02	2.884 (5)	176
O6—H3*O*···O3^vi^	0.96	2.26	2.920 (5)	125
O6—H3*O*···O4^vi^	0.96	2.33	3.068 (5)	133
C2—H2···O2^ii^	0.95	2.54	3.285 (5)	135

Symmetry codes: (i) *x*−1, *y*, *z*−1; (ii) −*x*+1, *y*−1/2, −*z*+1; (iii) *x*+1, *y*, *z*; (iv) *x*, *y*, *z*−1; (v) −*x*, *y*+1/2, −*z*+1; (vi) −*x*+2, *y*−1/2, −*z*+2.
